# Subsoiling Before Wheat Sowing Enhances Grain Yield and Water Use Efficiency of Maize in Dryland Winter Wheat and Summer Maize Double Cropping System Under One-Off Irrigation Practice During the Wheat Season

**DOI:** 10.3390/plants14050738

**Published:** 2025-02-28

**Authors:** Yanmin Peng, Kainan Zhao, Jun Zhang, Kaiming Ren, Junhao Zhang, Jinhua Guo, Rongrong Wang, Huishu Xiao, Peipei Jiang, Ninglu Xu, Ming Huang, Jinzhi Wu, Youjun Li

**Affiliations:** School of Agriculture, Henan University of Science and Technology, Luoyang 471023, China; pengym_1998@163.com (Y.P.); zhaokainan6@126.com (K.Z.); bjzhangjun@126.com (J.Z.); renkaiming1848@163.com (K.R.); zh15237517960@163.com (J.Z.); guojinhua0723@163.com (J.G.); wangrr934@163.com (R.W.); xiaohuishu9@126.com (H.X.); jiangpeipei202406@163.com (P.J.); xnl_lulu1129@163.com (N.X.); huangming_2003@126.com (M.H.)

**Keywords:** tillage methods, irrigation practices, summer maize, grain yield, water use efficiency, PLSPM, TOPSIS

## Abstract

The winter wheat and summer maize double cropping system is the primary cropping pattern for wheat and maize in dryland areas of China. The management of tillage in this system is typically conducted before wheat sowing. However, few studies have validated and quantified the impact of tillage methods before wheat sowing and irrigation practices during the wheat season on the yield formation and water use efficiency of summer maize. Therefore, this study hypothesized that subsoiling before wheat sowing improves maize yield and WUE by enhancing soil moisture retention and plant development. A three-year field experiment with a two-factor split-plot design was conducted at the junction of the Loess Plateau and the Huang-Huai-Hai Plain in China for validation, from 2019 to 2022. Three tillage methods before wheat sowing (RT: rotary tillage; PT: plowing, SS: subsoiling) were assigned to the main plots, and two irrigation practices during wheat growing season (W0: zero-irrigation; W1: one-off irrigation) were assigned to subplots. We measured the soil moisture, grain yield, dry matter accumulation, nitrogen (N), phosphorus (P), and potassium (K) accumulation, and water use efficiency of summer maize. The results indicated that subsoiling before wheat sowing increased soil water storage at the sowing of summer maize, thereby promoting dry matter and nutrient accumulation. Compared to rotary tillage and plowing, subsoiling before wheat sowing increased grain yield and water use efficiency of maize by an average of 19.5% and 21.8%, respectively. One-off irrigation during the wheat season had negative effects on pre-sowing soil water storage and maize productivity in terms of yield and dry matter accumulation. However, subsoiling before wheat sowing can mitigate these negative effects of one-off irrigation. Correlation analysis and path model results indicated that tillage methods before wheat sowing had a greater impact on soil water storage and maize productivity than irrigation practices during wheat growing season. The most direct factor affecting maize yield was dry matter accumulation, whereas the most direct factor affecting water use efficiency was nutrient accumulation. The technique for order preference by similarity to an ideal solution (TOPSIS) comprehensive evaluation indicated that subsoiling before wheat sowing was superior for achieving high maize yield and water use efficiency under the practice of one-off irrigation during the wheat season. These findings offer practical guidance for optimizing soil water use and maize productivity in drylands.

## 1. Introduction

Maize (*Zea mays* L.) is one of the most important food, feed, and economic crops globally, playing a crucial role in maintaining global food security and economic development [1,2,3]. In China, the winter wheat (*Triticum aestivum* L.) and summer maize (hereafter referred to as wheat–maize) double cropping system is the primary cropping pattern for wheat and maize. This system helps to enhance the utilization efficiency of resources such as light and temperature [4]. However, in this system, rotary tillage or no-till planting is generally adopted during the maize season for saving time and costs, which weakens the role of soil tillage in improving soil properties for high maize yields [5]. Research has shown that previous crop tillage has residual effects that can regulate soil properties for subsequent maize crops and increase yield [6,7]. Moreover, the wheat–maize double cropping system is an important pattern in the drylands [6]. In this system, water deficiency is the primary factor limiting crop yield, and the agronomic practice that help to improve water scarcity can strongly increase maize productivity [8,9]. In recent years, the progression of the High-Standard Farmland Construction Program in China and around the world has been swift, ensuring at least one-off irrigation (only irrigated once time during the wheat growth stage) in many dryland regions—arid, semi-arid, and semi-humid drought-prone—that previously lacked irrigation [8]. Previous studies have shown that one-off irrigation during the wheat season can significantly improve wheat yield and water use efficiency [8,9,10]. However, the limited irrigation (one-off irrigation belongs to this scope) during the wheat season may enhance soil water uptake by in-season crops, which may result in dry soil at the sowing and yield reduction of the subsequent maize [11]. Therefore, it is necessary to understand how one-off irrigation during the wheat season affects soil water storage before the sowing of maize and the nutrient accumulation, yield, and water use efficiency of maize, and seek some effective agronomic strategy to compensate for the water deficiency induced by one-off irrigation during the wheat growing season on maize productivity in the dryland wheat–maize double cropping system.

Optimized tillage methods can improve soil physio-chemical properties, thereby enhancing soil water retention capacity [12,13]. Nevertheless, reasonable tillage practices can harmonize the relationships among water, fertilizer, air, and heat in the soil, promote the root growth of maize, enhance the dry matter and nutrient accumulation and utilization in above-ground parts, and improve maize yield and water use efficiency [12,14,15]. An eight-year experiment in the semi-humid region of China demonstrated that, compared to deep plowing, rotary tillage before wheat sowing could loosen the surface soil and increase the moisture content in the deeper soil layers, thereby significantly enhancing the dry matter accumulation and grain yield of maize by 10.7–11.7% and 11.0–12.3%, respectively, in the wheat–maize double cropping system [16]. However, other studies have indicated that long-term rotary tillage before wheat sowing may cause the plow pan layer to become shallower, which restricts nutrient uptake by maize and is detrimental to maize growth [17,18]. Kuang et al. also demonstrated that deep tillage before wheat sowing improved soil properties, leading to the significant increases in the 100-kernel weight and grain yield of subsequent maize by 2.6–4.2% and 8.3–13.1%, respectively, compared to rotary tillage [6]. Similarly, Latifmanesh et al. found that deep tillage before wheat sowing increased yield by 8.0% and 4.4%, respectively, compared to rotary tillage and no-tillage [7]. Conservation tillage is beneficial for increasing soil water retention capacity, providing more water for crop growth [19,20]. Wang et al. found that, in this area, compared to traditional tillage, conservation tillage increased the bulk density of the surface soil but decreased the bulk density of the subsoil, leading to better water storage during irrigation and rainfall in winter wheat fields [21]. Subsoiling, as a conservation tillage method, has been demonstrated to easily create a structure with alternating loosened and compacted soil layers, optimizing soil moisture, fertility, aeration, and thermal regimes in the tillage layer, allowing roots to grow earlier and extend to deeper soil layers, facilitating the utilization of deep soil water and nutrients by crops [22,23,24]. Sun et al. indicated that subsoiling before wheat sowing decreased the soil bulk density in the 30–40 cm soil layer at summer maize maturity by 3.9%, and increased the soil porosity, root length density, and grain yield of maize by 5.9%, 30.0%, and 4.7%, respectively [25]. Other field studies showed that subsoiling before wheat sowing could break the plow pan, thus allowing water to penetrate deeper into the soil (below 60 cm) during the summer maize growth period, optimizing water supply for maize growth [6], increasing the areas of green leaf, delaying leaf senescence, and achieving higher dry matter accumulation and yield in maize [26].

Irrigation increases soil moisture and alleviates the adverse effects of drought stress on crop growth and increases crop yields [27,28]. Previous studied have shown that the irrigation practices can not only affecting on crop growth in the current season, but also affect the productivity of the subsequent crop in a rotation cropping system [29,30,31,32]. Previous studies have shown that limited irrigation during the wheat season increases soil water consumption, which was unfavorable for summer maize growth and significantly reduced maize yield in the wheat–maize double cropping system [29,33]. Conversely, higher residual soil moisture from full irrigation during the wheat season can enhance summer maize yield [34]. In the study area, one-off irrigation during the wheat season can delay wheat leaf senescence [10], allowing the crop to fully utilize soil water and nutrients in later growth stages, and increased wheat yield by 56%, but it leads to dry soil after harvest [8]. Indeed, under limited water supply conditions during the wheat season, water conservation measures can mitigate the negative effects of limited irrigation on summer maize growth [29]. Wei et al. indicated that the combination of subsoiling and proper irrigation can reduce evapotranspiration, increasing crop yield and water use efficiency by 34.2–84.1% and 19.5–54.3%, respectively [35]. However, few studies have validated and quantified the impact of tillage methods before wheat sowing and irrigation practices during the wheat season on the yield formation and water use efficiency of summer maize.

The technique for order preference by similarity to an ideal solution (TOPSIS) is usually used for comprehensive evaluation of different field management practices [36,37]. In this method, the pathway analysis using Partial Least Squares Path Modeling (PLSPM) is used for quantifying the causal relationships and interaction mechanisms among various variables to enhance scientific understanding of crop production [38]. Then, the TOPSIS method comprehensively evaluates each scheme by measuring the distance between each evaluation scheme and the ideal solution [36,39]. Previous studies have used PLSPM to establish a pathway model including crop rotation, soil chemical properties, and maize growth, showing that crop rotation had a limited impact on maize productivity through soil chemical properties [38,40,41]. Many studies have also used the TOPSIS method for the comprehensive evaluation of field management practices based on a series of different indicators [36,37]. They concluded that the best drought-resistant cultivation model for maize planting in the region is plastic film mulching [36], and the optimal irrigation period is root zone alternate irrigation during the seedling stage when mild water deficiency occurs [37]. However, the comprehensive evaluation of the impact of tillage methods before wheat sowing and irrigation practices during the wheat season on the yield formation and water use efficiency of summer maize using the TOPSIS method is still limited.

Therefore, we hypothesized that subsoiling before winter wheat sowing increases soil water storage before summer maize sowing under the practice of one-off irrigation during the wheat season, thereby enhancing the nutrient accumulation, yield, and water use efficiency of maize. We conducted a three-year split-plot field experiment (three tillage methods—rotary tillage, plowing, and subsoiling—before wheat sowing, and two irrigation practices—zero-irrigation and one-off irrigation—during the wheat growing season) in a typical semi-humid drought-prone region in China. This study aimed to assess how subsoiling before winter wheat sowing influences maize yield and WUE under the practice of one-off irrigation during the wheat season. The comprehensive analysis was performed using PLSPM and TOPSIS methods. If this performs well in this region, it could be extended to similar dryland regions where one-off irrigation is assured.

## 2. Results

### 2.1. Soil Water Storage

**Figure 1:**
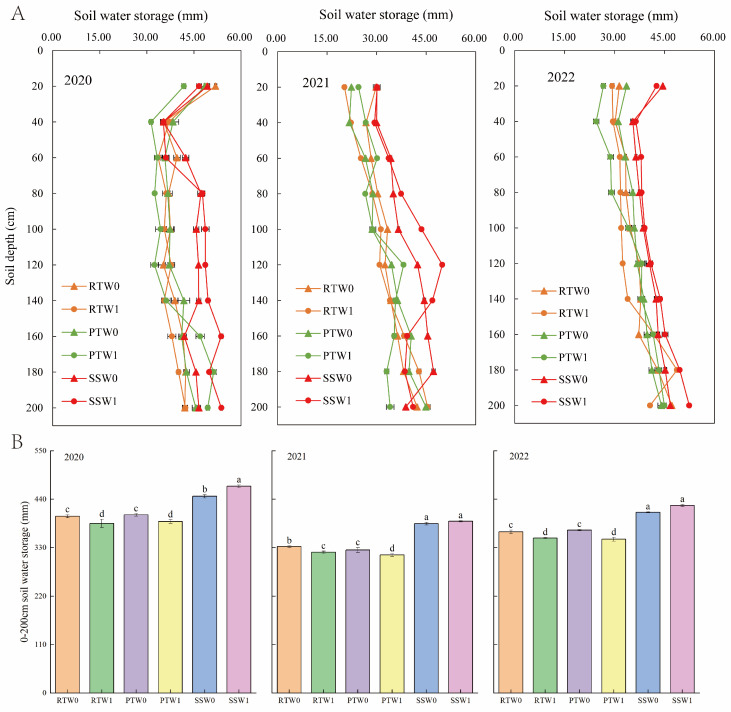
Effects of tillage methods before wheat sowing and irrigation practices during the wheat season on soil water storage in each soil layer (**A**) and total soil water storage in the 0–200 cm layer (**B**) before maize sowing. RT, PT, and SS represent rotary tillage, plowing, and subsoiling before wheat sowing, respectively. W0 and W1 represent zero-irrigation and one-off irrigation practice during the wheat season, respectively. Different lowercase letters indicate significant differences at *p* < 0.05.

Figure 1 shows that tillage methods before wheat sowing significantly affected soil water storage at the sowing of maize over the three years. Subsoiling before winter wheat sowing significantly increased soil water storage before summer maize sowing, particularly at depths of 60–140 cm (Figure 1A). Over the three years, the average soil water storage in the 0–200 cm layer under subsoiling was significantly increased by 17.2% and 17.5% compared to rotary tillage and plowing, respectively (Figure 1B). The impact of irrigation practices during the wheat season on soil water storage at the sowing of summer maize varied depending on the tillage methods before wheat sowing. Compared to W0, W1 decreased soil water storage in the 0–200 cm layer by 3.9% and 4.3%, respectively under RT and PT, whereas it increased by 3.6% under subsoiling. The treatment of subsoiling before wheat sowing and one-off irrigation during the wheat season (SSW1) increased maize pre-sowing soil water storage in the 80–140 cm soil layers compared to other treatments, especially when rainfall was severely deficient in June 2021. The soil water storage in the 0–200 cm layers under SSW1 was not significantly different from SSW0 in 2021 and 2022, whereas it was significantly higher than other treatments.

### 2.2. Above-Ground Dry Matter Accumulation

**Figure 2:**
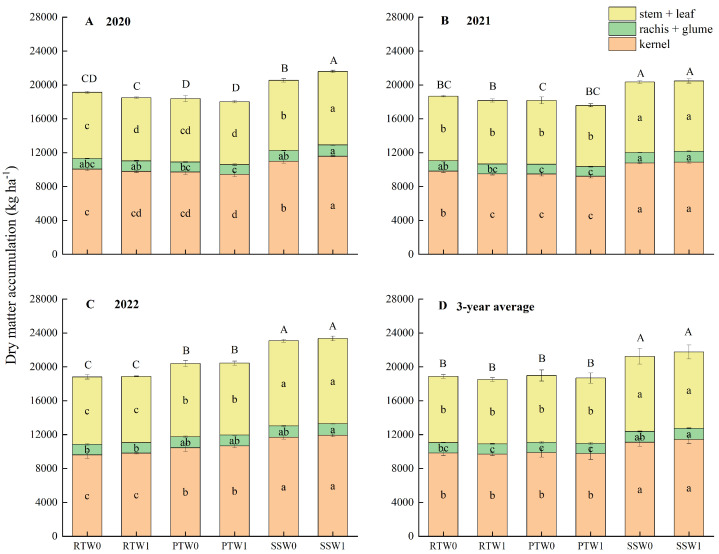
Effects of tillage methods before winter wheat sowing and irrigation practices during the wheat season on the dry matter accumulation of maize in 2020, 2021, 2022 and the three-year average. Note: RT, PT, and SS represent rotary tillage, plowing, and subsoiling before wheat sowing, respectively. W0 and W1 represent zero-irrigation and one-off irrigation during the wheat season, respectively. Different lowercase letters within the same organ indicate significant differences among treatments for organ dry matter accumulation at the *p* < 0.05 level. Different uppercase letters indicate significant differences among treatments for above-ground dry matter accumulation at the *p* < 0.05 level.

Figure 2 shows that subsoiling before wheat sowing increased the above-ground dry matter accumulation by 16.3% and 14.7%, respectively, compared to rotary tillage and plowing, in which the dry matter accumulation in stem + leaves and kernels under subsoiling were respectively 15.8% and 6.5% higher than to rotary tillage, and 15.0% and 8.7% higher than plowing. Although W1 had no significant difference in average dry matter accumulation from W0, the effects of irrigation practices on dry matter accumulation varied depending on the tillage method before wheat sowing. Compared to W0, W1 decreased dry matter accumulation under rotary tillage and plowing, while it increased under subsoiling. Particularly in 2020 when rainfall was insufficient, compared to W0, W1 decreased dry matter accumulation by 2.8% for kernels and by 5.2% for stem + leaf under rotary tillage, while they were significantly increased by 5.4% and 4.7% respectively under subsoiling. Overall, SSW1 obtained the highest above-ground dry matter accumulation, which had no significant difference from SSW0 except for 2020. However, over the three years, SSW1 significantly increased the above-ground dry matter accumulation by 14.6–17.6% compared to other treatments.

### 2.3. Above-Ground Nutrient Accumulation

**Figure 3:**
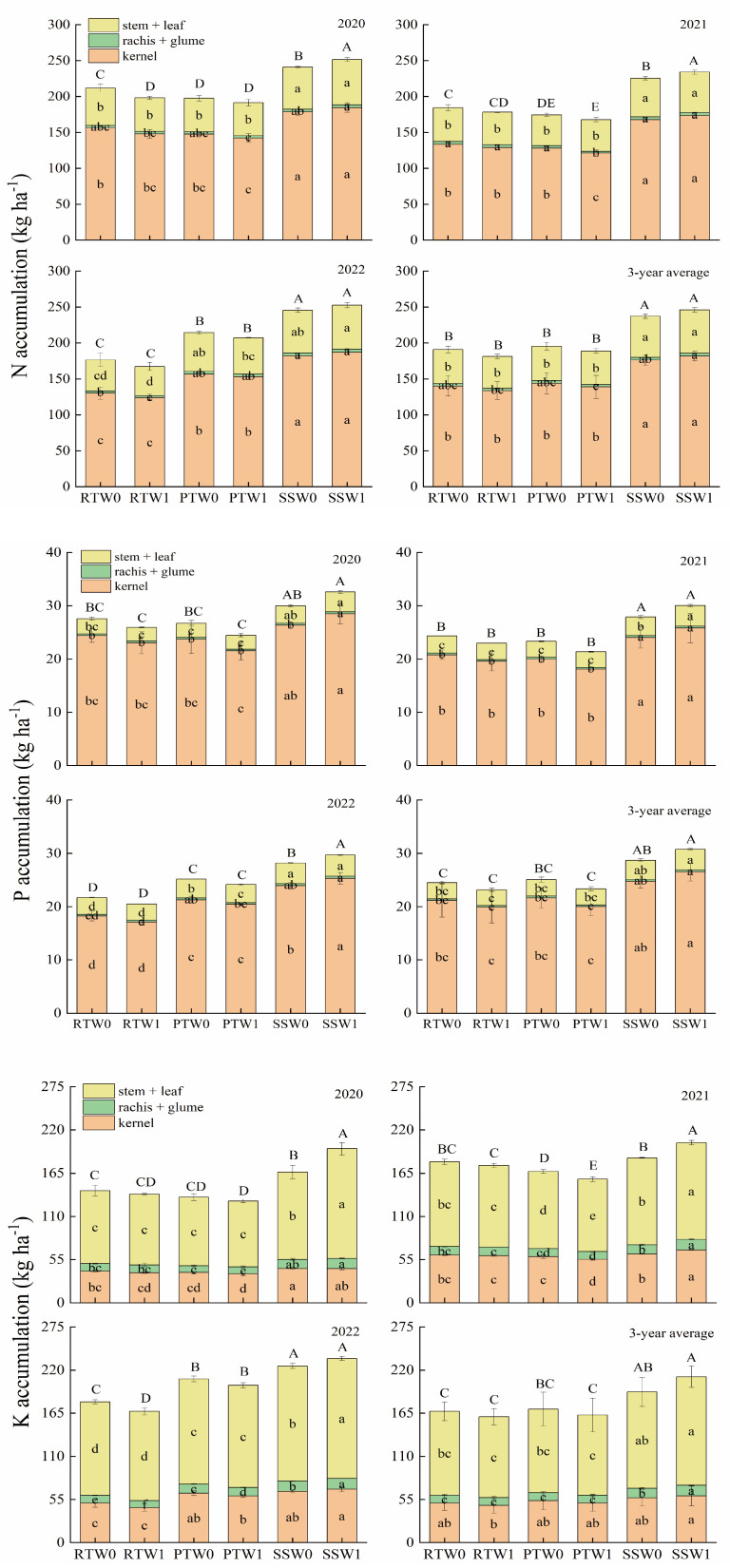
Effects of tillage methods before wheat sowing and irrigation practices during the wheat season on N, P and K accumulation of summer maize. Note: RT, PT, and SS represent rotary tillage, plowing, and subsoiling before wheat sowing, respectively. W0 and W1 represent zero-irrigation and one-off irrigation practice during the wheat season, respectively. Different lowercase letters within the same organ indicate significant differences among treatments for organ N, P and K accumulation at the *p* < 0.05 level. Different uppercase letters indicate significant differences among treatments for above-ground N, P and K accumulation at the *p* < 0.05 level.

Over the three years, subsoiling increased the above-ground nitrogen (N), phosphorus (P), and potassium (K) accumulation by 29.7%, 24.7%, and 23.2%, respectively, compared to rotary tillage, as well as by 25.6%, 22.8%, and 21.3% compared to plowing (Figure 3). On average, irrigation practices during the wheat season did not significantly affect the nutrient accumulation for N, P, and K in maize. However, the effectiveness of irrigation practices varied depending on the tillage method before wheat sowing. Compared to W0, W1 decreased nutrient accumulation under rotary tillage and plowing, whereas it was increased under subsoiling. Particularly, the kernel N, P, and K accumulation respectively decreased by 4.6%, 5.8%, and 5.8% under rotary tillage, and by 3.6%, 7.5%, and 5.5% under plowing, whereas it increased by 3.1%, 7.0%, and 4.6% under subsoiling. The interaction between tillage methods before wheat sowing and irrigation practice during the wheat growing season had a significant effect on above-ground nutrient accumulation in maize at maturity. Specifically, the N, P, and K accumulation under SSW1 were significantly increased by 30.2%, 28.3%, and 28.1%, respectively, compared to other treatments except for SSW0.

### 2.4. Grain Yield

Both tillage methods before wheat sowing and irrigation practices during the wheat season significantly affected the grain yield and kernels per ear of maize except for 2022 (Table 1). Compared to rotary tillage and plowing, subsoiling increased the kernels per ear by 3.8–25.4% and 4.6–23.2%, the 100-kernel weight by 3.2–10.8% and 7.5–16.4%, and the grain yield by 14.4–29.2% and 13.7–23.3%, respectively. Over the three years, compared to W0, W1 reduced the kernels per ear by 3.0% and 1.6%, 100-kernel weight by 4.2% and 2.6%, respectively under rotary tillage and plowing, while increased by 3.2% and 3.6% under subsoiling. The kernels per ear, 100-kernel weight, and grain yield under SSW1 were significantly higher than other treatments except for SSW0.

### 2.5. ET and Water Use Efficiency

Table 2 showed that tillage methods before wheat sowing significantly affected evapotranspiration (ET) during the maize season and water use efficiency (WUE) of maize expect for 2020. On average, compared to rotary tillage, subsoiling did not affect ET, but significantly increased WUE by 20.0%. Compared to plowing, subsoiling significantly reduced ET by 3.0% and increased WUE by 23.6%. Overall, irrigation practices during the wheat season had no significant impact on ET and WUE over the three years. However, compared to W0, W1 tended to decrease WUE under rotary tillage and plowing, whereas increased WUE under subsoiling. Although there was no interaction effect of tillage methods before wheat sowing and irrigation practices during the wheat season on ET, SSW1 increased WUE by 2.0–22.7%, with an average of 18.1%, compared to other treatments.

### 2.6. Correlation and Path Model

#### 2.6.1. Correlation Analysis

**Figure 4:**
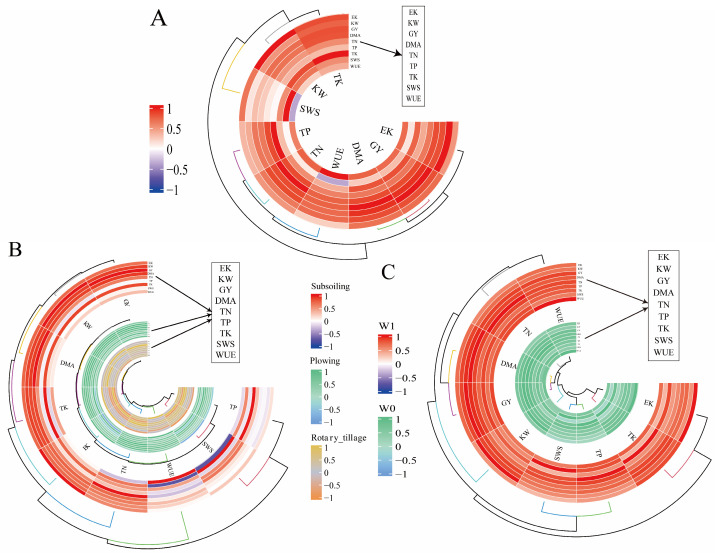
Relationships among the SWS, grain yield, yield components, dry matter accumulation, nutrient accumulation, and water use efficiency under the combination of tillage methods before wheat sowing and irrigation practices during the wheat season (**A**); tillage methods before wheat sowing (**B**) and irrigation practices during the wheat season (**C**).

Figure 4 showed that there were correlations between every two indicators in term of yield components, plant dry matter accumulation, nutrient accumulation, pre-sowing soil water storage, and yield and water use efficiency in most cases. However, these correlations varied depending on tillage methods before wheat sowing and irrigation practices during the wheat season. The main difference laid in the relationship between soil water storage at sowing and water use efficiency, with the negative correlation under the interactions of tillage methods before wheat sowing and irrigation practices during the wheat season (Figure 4A), and under different tillage methods before wheat sowing (Figure 4B). However, there was a positive correlation between soil water storage at sowing and water use efficiency under irrigation practices during the wheat season (Figure 4C).

#### 2.6.2. Partial Least Squares Path Analysis

**Figure 5:**
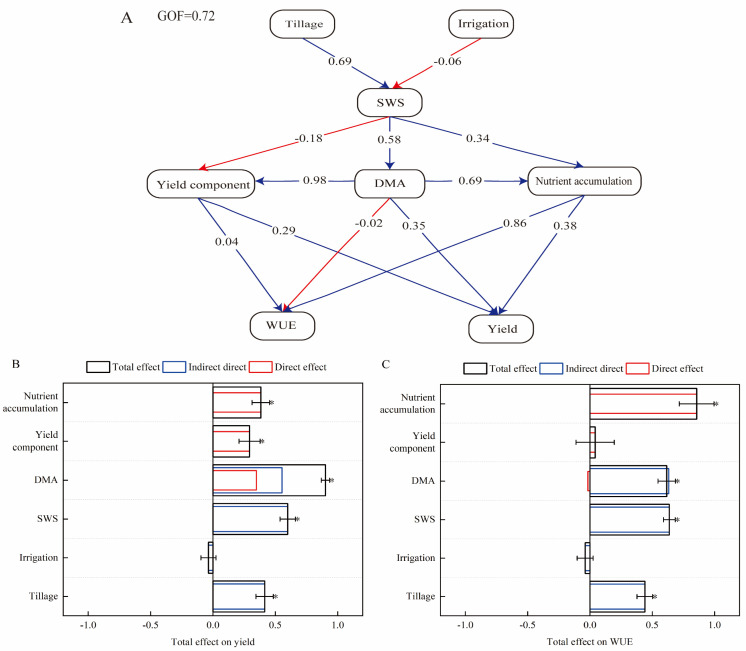
Partial least squares path model (**A**), standardized total effects on grain yield (**B**), and water use efficiency (**C**) based on the model. Note: Path coefficients were calculated after 1000 bootstrap repetitions and are reflected by the width of the arrows, with blue and red indicating positive and negative effects, respectively. The model was evaluated using the goodness-of-fit statistic, and its value was 0.72. * Indicates significance at *p* < 0.05.

To further determine the complex relationships among pre-sowing soil water storage, yield traits, dry matter accumulation, nutrient accumulation, and the water use efficiency of maize under different treatments, a partial least squares path model was established (Figure 5A). The goodness-of-fit value was 0.72, indicating a good model fit. These results showed that both tillage methods before wheat sowing and irrigation practice during maize season significantly affected soil water storage at the sowing of maize; greater effectiveness was obtained under tillage methods before wheat sowing than under irrigation practice during maize season. Soil water storage at the sowing of maize directly positively affected yield components and nutrient accumulation. Yield components and nutrient accumulation also had significant positive effects on yield and water use efficiency. Dry matter accumulation had a positive effect on yield but negative effect on water use efficiency. Overall, the model indicated that maize yield was primarily influenced by dry matter accumulation (Figure 5B), and water use efficiency was mainly influenced by nutrient accumulation (Figure 5C).

### 2.7. Comprehensive Evaluation

#### 2.7.1. Determination of Weight Using the Entropy Method

The weights of entropy method for yield, kernels per ear, 100-kernel weight, dry matter and N, P, and K accumulation, soil water storage at sowing, and water use efficiency of maize were positive, indicating that the aforementioned indexes were positive indicators. Their weights for 2020 were 0.192, 0.090, 0.090, 0.131, 0.107, 0.066, 0.124, 0.103, and 0.098; for 2021, they were 0.131, 0.064, 0.099, 0.095, 0.129, 0.138, 0.080, 0.156, and 0.108; and for 2022, they were 0.153, 0.080, 0.081, 0.083, 0.070, 0.084, 0.059, 0.273, and 0.116, respectively.

#### 2.7.2. TOPSIS Comprehensive Evaluation

Table 3 indicates that the comprehensive evaluation value of RTW1 was significantly lower than that of RTW0, and that of PTW1 was also significantly lower than that of PTW0. However, the comprehensive evaluation value of SSW1 was significantly higher than that of SSW0. Under SSW1, the comprehensive values of each indicator were closer to the positive ideal solution and farther from the negative ideal solution. The three-year comprehensive evaluation value of SSW1 was significantly higher than other treatments by 12.8–342.9%. Especially in 2020, when rainfall was insufficient, the comprehensive evaluation value of SSW1 was the highest at 0.93. These results suggested that subsoiling before wheat sowing under one-off irrigation during the wheat season better aligns with the goals of high yield and high efficiency of maize in the wheat–maize double cropping system.

## 3. Discussion

### 3.1. Soil Water Storage Affected by Subsoiling Before Wheat Sowing Combined with One-Off Irrigation During the Wheat Season

In the double cropping systems, tillage managements for the previous crop have residual effects on soil environment of the subsequent crop [6,7,26]. Previous studies have shown that subsoiling before wheat sowing can reduce soil bulk density and soil penetration resistance during maize growth [42], increase soil porosity, and promote the infiltration of irrigation water and rainfall into the soil [6,7]. Our study also indicated that, compared to rotary tillage and plowing, subsoiling before wheat sowing significantly increased the total water storage in the 0–200 cm soil layer by 17.2% and 17.5%, respectively. The reason for this difference was that subsoiling increased water storage in the 60–140 cm soil layers (Figure 2). This was mainly because subsoiling before winter wheat sowing promotes wheat root growth [43,44], thus the subsoiling-induced dense root system enhances the connectivity among soil pores, guiding and promoting deeper soil water infiltration [6,45]. In addition, subsoiling increases soil stability [46], thus decreasing the evaporation rate of soil moisture from deeper layers [47].

**Figure 6:**
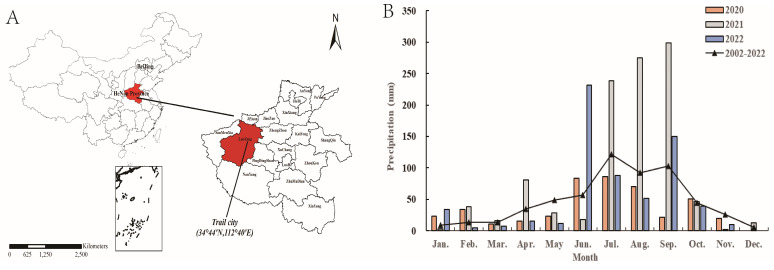
Location (**A**) and precipitation (**B**) of the experimental field in 2020–2022.

Many studies reported that soil water storage before sowing of the subsequent crop was affected by the irrigation amount and water consumption during the previous crop season [35,48,49]. Some studies showed that the limited irrigation increased water consumption during the wheat season, leading to dry soil before maize sowing [11,33,34]. However, Fang et al. found that increasing irrigation amount during the wheat season increased soil water storage before the sowing of subsequent maize [29]. In our trial, one-off irrigation during the wheat season resulted in the soil storage at the sowing of maize being 5.17 mm lower on average than zero-irrigation over three years. However, the soil water storage change induced by one-off irrigation varied depending on tillage methods before wheat sowing. It decreased by 3.9% and 4.3% under rotary tillage and plowing, respectively, but increased by 3.6% under subsoiling. This was mainly because subsoiling increased soil water holding capacity, enhancing soil water storage during the wheat season [50,51]. Additionally, the results of this trial indicated that tillage methods before wheat sowing had a stronger impact on soil water storage at the sowing of maize than irrigation practices during the wheat season (Figure 6). This may be due to the well-done water protection ability of subsoiling and the limited irrigation amount in our trials (<45 mm). Zhang et al. also indicated that soil storage reduction due to water-saving irrigation can be partially offset by subsoiling [52]. In conclusion, although the coupling effect of tillage methods before wheat sowing and irrigation practices during the wheat season was only significant in some years, the combination of subsoiling before wheat sowing and one-off irrigation during the wheat season increased soil water storage at the sowing of maize, particularly in the 80–140 cm soil layers when rainfall was insufficient (Figure 2).

### 3.2. Dry Matter and Nutrient Accumulation Affected by Subsoiling Before Wheat Sowing Combined with One-Off Irrigation During the Wheat Season

Tillage methods alter soil compaction and other properties, thus in turn affecting soil moisture, crop growth and the accumulation of dry matter and nutrients [25,53,54]. As our study showed, compared to rotary tillage and plowing, subsoiling before wheat sowing significantly increased dry matter and N, P, and K accumulation in maize in the double cropping system. This was because subsoiling before wheat sowing can help to break the plow pan in fields [6], promote vertical root distribution in maize, and form a stronger root system, thereby facilitating water and nutrient absorption [55,56], enhancing photosynthesis, and leaf antioxidant characteristics during the grain filling period, ultimately achieving higher dry matter and nutrient accumulation [57,58]. Additionally, our study also indicated a significant correlation between soil moisture before maize sowing and dry matter accumulation. This was mainly ascribed to the soil moisture and other properties improvement induced by subsoiling not only promoting maize root growth but also improving nutrient uptake and accumulation by plants. Particularly, the deep soil moisture improvement under subsoiling plays a critical role in nutrient uptake during the grain filling period, thereby enhancing maize dry matter accumulation [59].

Previous studies have shown that water stress during the maize growth stage disrupted the photosynthetic membrane, reduced chlorophyll content in leaves, and lowered dry matter and nutrient accumulation [60,61]. In this study, compared to zero-irrigation, one-off irrigation tended to decrease the dry matter and nutrient accumulation of maize. Increased water storage through optimizing agronomic measures can mitigate the negative impact of soil water deficiency on the dry matter and nutrient accumulation of maize [29]. Our study showed that one-off irrigation decreased the dry matter and nutrient accumulation under rotary tillage and plowing, but increased the dry matter, N, P, and K accumulation by 5.4%, 3.1%, 7.0%, and 4.6%, respectively, under subsoiling compared to zero-irrigation. These results indicated that subsoiling can mitigate the negative impact of one-off irrigation during the wheat season on maize dry matter and nutrient accumulation.

### 3.3. Yield and Water Use Efficiency of Maize Affected by Subsoiling Before Wheat Sowing Combined with One-Off Irrigation During the Wheat Season

Kernels per ear and 100-kernel weight directly affect maize yield [62,63]. This study indicated that subsoiling during the wheat season significantly increased kernels per ear and 100-kernel weight compared to rotary tillage and plowing, leading to an increase in yield by 19.5% and 19.5%, respectively. Additionally, this study also found that, one-off irrigation during the wheat season had a significant impact on maize yield and kernels per ear except for 2022, however, it had no significant effect on 100-kernel weight over the three years. This was mainly because irrigation during the wheat season significantly affected soil moisture at the sowing of maize [29,33], and this soil water change can not only regulate the nutrient uptake and photosynthetic rate during the early growth stages [19,64], but also regulate the extension of the pollen tubes, pollination rate, and kernels per ear [65,66], thus affecting the crop growth, and yield formation of maize.

Crop water use efficiency (WUE) is determined by water consumption during the growing period (evapotranspiration) and yield at maturity [62,67]. In our trial, rotary tillage, plowing, and subsoiling before wheat sowing had no significant impact on water consumption during the summer maize growth period. Kuang et al. also showed that rotary tillage and subsoiling during the wheat season had no significant impact on soil water consumption during the maize season [6]. Therefore, in our trails, WUE is only related to yield, and the subsoiling before wheat sowing significantly improved soil water storage before summer maize sowing, which increased maize yield, resulting in a 20.0% and 23.6% increase in WUE compared to rotary tillage and plowing, respectively. The findings confirmed our hypothesis that subsoiling enhanced WUE. However, this result was inconsistent with the field experiment for winter wheat–summer maize system in the North China Plain, where subsoiling increased soil water consumption during the maize season [61].

### 3.4. Pathway Analysis Using PLSPM and Comprehensive Evaluation

Many studies have shown a significant synergistic effect between soil moisture and maize production [6,68]. However, our correlation analysis showed that the correlation was negative between soil water storage at sowing and water use efficiency of maize under the interaction of the combination of tillage methods before wheat sowing and irrigation practices during the wheat season. This result aligns with correlation analyses under different methods before wheat sowing. The reason maybe that higher soil water storage at sowing leads to shallower maize roots, which is unfavorable for yield formation, thus reducing water use efficiency, especially under drought conditions [69,70].

Previous studies used PLSPM to establish a pathway model including crop rotation, soil chemical properties, and maize growth, showing that crop rotation had a limited impact on maize productivity through soil chemical properties [38,40,41]. The results of our study indicated that tillage methods before wheat sowing and irrigation practices during the wheat season affected maize yield by influencing soil water storage at the sowing of maize. The soil water storage at the sowing of maize had a higher effect on dry matter and nutrient accumulation than on yield components. Therefore, despite the soil water storage at the sowing of maize was negatively correlated with yield components, it was ultimately positively correlated with yield and water use efficiency. The impact of tillage methods before wheat sowing and irrigation practices during the wheat season on yield was mainly through the effect of soil water storage on dry matter accumulation, while the impact on water use efficiency was primarily through nutrient accumulation. Overall, the effect of tillage methods before wheat sowing was much greater than that of irrigation practices during the wheat season, explaining the different impacts of one-off irrigation on maize production under different tillage methods.

TOPSIS has the issue of incomparability among multiple indicators due to different dimensions [39]. Researchers tend to combine the entropy weight method with TOPSIS for comprehensive evaluation of multiple objectives. Previous studies used the entropy weight method and TOPSIS to comprehensively evaluate the drought resistance measures for increasing maize yield [36] and the effects of alternating root-zone irrigation on maize productivity at different growth stages [37]. Our study also used a comprehensive evaluation model combining the entropy weight method and TOPSIS to evaluate the effects of tillage methods before wheat sowing and irrigation practices during the wheat season on soil water storage at sowing, productivity, and water use efficiency of maize. The results indicated that the comprehensive value of SSW1 was the highest, further confirming that subsoiling before wheat sowing is the optimal field management practice for achieving high-yield and high-efficiency production of maize under the one-off irrigation practice during the wheat season in the wheat–maize double cropping season. In addition, conducting quantitative analysis and comprehensive evaluation of the relationship between soil moisture and crop growth under the given experimental conditions inevitably limits the broad applicability of our findings. Thus, if subsoiling before wheat sowing performs well on maize productivity in this region, it could be extended to similar dryland regions where one-off irrigation during the wheat season is assured.

The economic feasibility is a very important index to evaluate the agronomic strategies with different input. Subsoiling requires deeper soil penetration and powerful machinery, which generally leads to higher energy consumption compared to rotary tillage and plowing [71]. This higher energy input may pose economic and environmental challenges. Although previous studies have shown that subsoiling significantly improves crop economic benefits [50], if the subsoiling operation methods and energy efficiency cannot be continuously optimized, its long-term application may be limited. In addition, Repeated subsoiling may alter soil compaction and nutrient cycling over time. Although subsoiling has shown significant short-term soil improvement effects, its long-term sustainability needs further discussion [72]. Therefore, future studies should focus on how to maintain the economic feasibility and long-term sustainability of subsoiling before wheat sowing under the practice of one-off irrigation during the wheat season. For example, adopting more efficient machinery or renewable energy sources, and optimizing the timing and frequency of subsoiling operations are possible solutions. Additionally, integrating subsoiling with other sustainable agricultural practices, such as conservation tillage methods or biodiversity enhancement measures, should be considered. This study also showed that, the adoption of alternating rotary tillage and subsoiling can improve maize yield and water use efficiency of maize. Increasing the intervals of rotary tillage, such as two years of rotary tillage followed by one year of subsoiling or three years of rotary tillage followed by one year of subsoiling, maybe further enhance its long-term sustainability. However, further experimental validation for the proper intervals is needed for specific regions.

## 4. Materials and Methods

### 4.1. Experimental Site Description

The present experiment was conducted at Dugou Village, Yichuan County, Henan Province, China (Figure 6A), from October 2019 to October 2022. The experimental site is located in a typical semi-humid drought-prone area with a temperate continental monsoon climate. The average annual temperature is 13.9 °C, and the average annual sunshine duration is 2311 h. The average annual rainfall is 570.8 mm, with approximately 60% concentrated between June and September. During the maize growing seasons from 2020 to 2022, the total rainfall was 263.0 mm, 831.0 mm, and 521.6 mm, respectively (Figure 6B). The soil was classified as heavy loam with pH of 7.6, field capacity of 24.5%, bulk density of 1.33 g·cm^−3^, organic matter content of 12.4 g kg^−1^, total N content of 1.08 g kg^−1^, available P content of 12.7 mg kg^−1^, and available K content of 177.1 mg kg^−1^ in the 0–20 cm soil layer at the beginning of the experiment in 2019.

### 4.2. Experimental Design and Field Managements

The experiment employed a two–factor split-plot design. The main plots included three tillage methods: rotary tillage (RT, with a depth of 15 cm), plowing (PT, with a depth of 30–35 cm), and subsoiling (SS, with a depth of 35–40 cm) before wheat sowing. The depth of specific tillage was set based on the common practice of large-scale households in the study area. The subplots included two irrigation practices: one-off irrigation (W1) and zero-irrigation (W0) during the wheat season. After plowing and subsoiling, a rotary tiller was used to smooth the soil surface. Rotary tillage and plowing were conducted annually, while subsoiling was conducted biennially (subsoiling in 2019 and 2021, rotary tillage in 2020).

A one-off irrigation approach was selected based on the production practice and the results of our previous studies [8,10]. According to the results of previous studies and our filed experiment [8,10], we set the irrigation threshold: irrigation was replenished to 85% of field capacity when the soil moisture content in the 0–40 cm layer first fell below 60% of the field capacity after regreening of wheat. The irrigation amount was calculated according to the method of Ekrenel. [73]:

$$IA=10\times \rho b\times H\times \left(\beta i-\beta j\right)$$

where IA is the irrigation amount; ρb is the average bulk density (g·cm^−3^); H is the average depth of the planned wetting layer (cm); βi is the target soil moisture content (%); βj is the average soil moisture content before irrigation.

The irrigation amount was controlled by a water meter, and the irrigation date was 25 February 2020, 25 February 2021, and 1 March 2022. The irrigation amounts for RT, PT, and SS were 41.1 mm, 43.2 mm, and 37.8 mm in 2020; 39.7 mm, 42.1 mm, and 38.3 mm in 2021; and 37.2 mm, 40.1 mm, and 39.4 mm in 2022. Before summer maize sowing, N fertilizer of 210 kg N ha^−1^, P fertilizer of 90 kg P_2_O_5_ ha^−1^, and K fertilizer of 60 kg K_2_O ha^−1^ were evenly applied by hand. After that, a rotary tiller seeder (2BYF-3, Henan Changge Jinfenggu Agricultural Machinery Co., Ltd., Zhengzhou, China) was used for sowing immediately. The N, P, and K fertilizers were urea (N, 46%), triple superphosphate (P_2_O_5_, 12%), and potassium sulfate (K_2_O, 50%), respectively. The maize variety Zhongke Yu 505 was used. The planting density for summer maize was 6 × 10^4^ plants per hectare, with a row spacing of 60 cm and a plant spacing of 27.5 cm. Maize was sown in early and middle June, and harvested in late September or early October. There was no irrigation during the summer maize growing season. Other management practices, such as weeding and pest control, were conducted according to local practices.

### 4.3. Measurements and Methods

#### 4.3.1. Soil Water

Soil water content was measured by the oven-drying method. At sowing and harvest of maize in 2020–2022, soil samples were randomly collected from each plot at depth ranging from 0 to 200 cm, with increments of 20 cm, using an auger with a diameter of 4.0 cm. Soil samples from the same depth within each plot were thoroughly mixed. Fresh soil samples weighing 50 g ± 5 g was placed in aluminum boxes and oven-dried at 105 °C for 24 h to measure soil moisture content.

#### 4.3.2. Grain Yield and Yield Components

At harvest, ears of the sample area with 1.8 m × 2.2 m were manually collected from the center of each plot. Then 10 representative ears were selected to count the number of rows per ear and the number of kernels per row, calculating the kernels per ear. Following harvest, the ears were air-dried, threshed, and the grain obtained was weighed. To accurately determine grain moisture content and dry weight, 100-kernel samples of the air-dried grain were further oven-dried at 90 °C for 30 min and then at a reduced temperature of 65 °C for a duration of 24 h. Grain yield calculations for each plot were standardized to a uniform moisture content of 12.5%, using the air-dried grain weight and its determined water content to adjust the figures according to the method described by Echarte et al. [74].

#### 4.3.3. Dry Matter and Nutrient Accumulation

At maturity of maize in 2020−2022, 10 maize plants were cut from three different rows in each plot. To minimize bias caused by sample collection, these 10 representative plants were sampled within consistent growth conditions, and 3–4 plants from each row. After cutting off the root, samples were separated into three components in terms of stem + leaf, rachis + glume and grain. Sub-samples were oven dried at 105 °C for 30 min and then at 80 °C to determine the water contents and dry weight in each organ. Then, the oven-dried samples of grain, stem + leaf, and rachis + glume were ground with a ball miller (MM400, RETSCH, Haan, Germany), and then digested with H_2_SO_4_−H_2_O_2_. According to the method of Huang et al. [75], the N and P concentrations in the digest solution were determined using an Auto Analyzer 3 (AA3, Seal Company, Norderstedt, Germany) and K concentration was measured using a flame spectrophotometer (Flame Photometer 410, Sherwood Company, Bury, England). The nutrient (N, P, and K) accumulation in each organ were calculated as the dry weight (kg ha^−1^) multiplied by the corresponding nutrient concentration (g kg^−1^), and the dry matter, and nutrient accumulation in above-ground parts (kg ha^−1^) were calculated from the summed by each organ.

#### 4.3.4. Water Use Efficiency

Soil water storage (SWS), evapotranspiration, and water use efficiency was calculated according to Li [76]:

SWS=10×D×H×W

where SWS is the soil water storage in the 0–200 cm soil layer (mm); D is the soil bulk density (g·cm^−3^); H is the soil layer thickness (cm); W is the soil moisture content (%).

ET=P+U−R−F−∆W

where ET is the evapotranspiration during the growing period; P is the precipitation during the maize growing period (mm); R is the runoff (mm); U is the groundwater recharge (mm); F is the deep percolation (mm); ΔW is the difference in soil water storage between the 0–200 cm soil layer before maize sowing and after harvest (mm). When the groundwater table is deeper than 2.5 m, the U value can be ignored. In this experiment, the groundwater was 5 m deep, so all the values for U, F, and R were 0.

WUE=Y/ET

where Y is the grain yield (kg ha^−1^), and ET is the evapotranspiration during the maize growing season (mm).

### 4.4. Calculation of Comprehensive Evaluation Value

#### 4.4.1. Determination of Indicator Weights Using the Entropy Method

Since the evaluation indicators, such as yield and yield components, dry matter accumulation, nutrient accumulation, and water use efficiency are different and cannot be compared directly, the measured data for each indicator were normalized to eliminate the influence of different dimensions. The entropy weight method was then used for objective weight analysis, referring to the method of Zou et al. [77].

#### 4.4.2. Calculation of Comprehensive Evaluation Values for Each Scheme Using TOPSIS

The TOPSIS method evaluates each scheme by measuring the distance to the ideal solution. The best scheme is the one that is closest to the optimal solution and furthest from the worst solution. The comprehensive evaluation value of each treatment is represented by Ci (0 < Ci < 1), where a value closer to 1 indicates that the scheme is more conducive to high maize yield.

### 4.5. Statistical Analysis

The data were processed using Microsoft Excel 2016 (Microsoft Corp, Redmond, WA, USA) and SPSS 23.0 (SPSS Inc., Chicago, IL, USA) software, and analyzed using ANOVA and post-hoc Duncan’s test (*p* < 0.05). Figures were prepared using with Origin 2022 (Origin Lab, Northampton, MA, USA) software. Additionally, R 4.4.1 (R Core Team, University of Auckland, Auckland, New Zealand) was used for Partial Least Squares Path Modeling [78], with validation via the “plspm” package in R (1000 bootstrap repetitions). In this model, we defined pre-sowing soil water storage (SWS), dry matter accumulation (DMA), yield, and water use efficiency as observed variables. Yield components (including kernels per ear and 100-kernel weight) and nutrient accumulation (including N, P, and K accumulation) were defined as latent variables, while tillage and irrigation were considered dummy variables (Figure 6). The direct effects (path coefficients) indicate the direction and strength of the linear relationships between variables [69].

## 5. Conclusions

This study demonstrated that subsoiling before wheat sowing significantly increased soil water storage at the sowing of maize compared to rotary tillage and plowing, thereby enhancing maize dry matter and nutrient accumulation in above-ground parts, ultimately increasing yield and water use efficiency of maize by 19.5% and 21.8%, respectively. One-off irrigation during the wheat season decreased dry matter and nutrient accumulation, and grain yield of maize, compared to zero-irrigation. Subsoiling before wheat sowing can mitigated these negative effects. Partial least squares path analysis indicated that the impact of tillage methods before wheat sowing and irrigation practices during the wheat season on yield was primarily through the effect of soil water storage on dry matter accumulation, while their influence on water use efficiency was mainly through nutrient accumulation. Furthermore, the TOPSIS comprehensive evaluation results indicated that subsoiling before wheat sowing is an alternative strategy for achieving high-yield and high-efficiency of maize under the practice of one-off irrigation during the wheat season in the wheat–maize double cropping system, it should be a recommended field management practice for the wheat–maize double cropping system in the region where one-off irrigation is assured during the wheat season. These results confirm our hypothesis that subsoiling before wheat sowing significantly enhances maize yield and WUE under one-off irrigation during the wheat season, future studies should focus on the multi-year effects of subsoiling on long-term sustainability in term of economic feasibility, yield stability, and soil prosperities.

## Figures and Tables

**Table 1:** Effects of tillage methods before wheat sowing and irrigation practices during the wheat season on yield and yield components of maize.

Treatments	Grain Yield (kg ha^−1^)				Kernels Per Ear (kernels ear^−1^)				100-Kernel Weight (g)			
	2020	2021	2022	Average	2020	2021	2022	Average	2020	2021	2022	Average
RTW0	9750 e	9564 b	9474 c	9596 b	454 cd	535 c	514 b	501 b	31 ab	30 bc	33 bc	31 bc
RTW1	9315 f	9239 b	9360 c	9305 b	438 e	526 d	516 b	493 b	31 b	29 bcd	32 c	31 bc
PTW0	9018 c	9024 bc	10,906 d	9649 b	462 c	529 cd	531 b	507 ab	28 c	28 cd	34 bc	30 c
PTW1	8664 d	8554 c	10,501 d	9239 b	446 de	524 d	513 b	494 b	29 c	27 d	33 bc	30 c
SSW0	10,643 b	10,653 a	12,038 a	11,111 a	541 b	544 b	581 a	555 ab	32 ab	31 ab	36 a	33 ab
SSW1	11,160 a	10,944 a	12,297 a	11,467 a	578 a	557 a	589 a	575 a	32 a	33 a	36 a	34 a
Tillage (T)	361.7 **	66.3 **	12.7 **	11.4 **	523.1 **	46.5 **	45.0 **	6.4 *	37.6 **	15.5 **	40.0 **	9.8 **
Irrigation (I)	46.7 **	6.0 *	3.3	0.9	51.5 **	19.0 **	1.3	0.6	0.1	3.6	2.5	1.0
T * I	0.5	0.1	0.3	0.0	4.4 *	1.2	0.7	0.0	2.3	0.4	0.0	1.0

Note: RT, PT, and SS represent rotary tillage, plowing, and subsoiling before wheat sowing, respectively. W0 and W1 represent zero-irrigation and one-off irrigation practice during the wheat season, respectively. Different lowercase letters following the data in the same column indicate significant differences among treatments at the *p* < 0.05 level. * and ** indicate significant variance at the *p* < 0.05 and *p* < 0.01 levels, respectively.

**Table 2:** The effects of tillage methods before wheat sowing and irrigation practices during the wheat season on ET during the maize season and water use efficiency of maize.

Treatments	Evapotranspiration (ET, mm)				Water Use Efficiency (kg ha^−1^ mm^−1^)			
	2020	2021	2022	Average	2020	2021	2022	Average
RTW0	454.7 a	433.7 a	471.7 c	453.4 a	21.4 b	22.0 b	20.1 b	21.2 b
RTW1	444.8 a	438.4 a	469.6 c	450.9 a	21 bc	21.1 bc	19.9 b	20.7 bc
PTW0	442.3 a	439.8 a	516.2 a	466.1 a	20.4 c	20.5 cd	21.1 b	20.7 bc
PTW1	443.7 a	440.8 a	498.7 b	461.1 a	19.5 d	19.4 d	21.1 b	20.0 c
SSW0	451.5 a	413.8 b	475.7 c	447.0 a	23.6 a	25.7 a	25.3 a	24.9 a
SSW1	459.1 a	415.7 b	483.4 c	452.7 a	24.3 a	26.3 a	25.4 a	25.4 a
Tillage (T)	2.4	17.5 **	39.5 **	0.3	126.3 **	127.3 **	67.5 **	223.5 **
Irrigation (I)	0	0.4	1.3	0	52.87	2.4	0.0	1.5
T * I	1.3	0.1	4.3 *	0.1	5.3 *	2.8	0.0	3.3

Note: RT, PT, and SS represent rotary tillage, plowing, and subsoiling before wheat sowing, respectively. W0 and W1 represent zero-irrigation and one-off irrigation practice during the wheat season, respectively. Different lowercase letters following the data with the same column indicate significant differences among treatments at the *p* < 0.05 level. * and ** indicate significant variance at the *p* < 0.05 and *p* < 0.01 levels, respectively.

**Table 3:** The degree of fit and ranking under different treatments for summer maize by TOPSIS method.

Treatments	2020				2021				2022			
	di^+^	di^−^	di	Ranking	di^+^	di^−^	di	Ranking	di^+^	di^−^	di	Ranking
RTW0	0.25	0.12	0.33 c	3	0.23	0.12	0.34 c	3	0.31	0.08	0.21 d	5
RTW1	0.29	0.09	0.23 d	4	0.28	0.08	0.22 d	4	0.35	0.04	0.11 e	6
PTW0	0.28	0.07	0.21 d	5	0.28	0.07	0.21 d	5	0.26	0.14	0.34 c	3
PTW1	0.31	0.04	0.12 e	6	0.32	0.03	0.08 e	6	0.33	0.09	0.22 d	4
SSW0	0.11	0.25	0.7 b	2	0.11	0.26	0.71 b	2	0.09	0.31	0.78 b	2
SSW1	0.03	0.33	0.93 a	1	0.05	0.31	0.86 a	1	0.05	0.36	0.88 a	1

Note: di^+^: The distance of each evaluation scheme to the positive ideal solution; di^−^: The distance of each evaluation scheme to the negative ideal solution; Ci: Closeness coefficient. Different lowercase letters following the data in the same column indicate significant differences among treatments at the *p* < 0.05 level.

## Data Availability

This study includes all supporting data, which can be obtained from the corresponding authors upon request.
